# Factors Associated with Community Participation among Individuals Who Have Experienced Homelessness

**DOI:** 10.3390/ijerph120911364

**Published:** 2015-09-10

**Authors:** Feng-Hang Chang, Christine A. Helfrich, Wendy J. Coster, E. Sally Rogers

**Affiliations:** 1 Graduate Institute of Injury Prevention and Control, Taipei Medical University, Taipei 11031, Taiwan; 2 Occupational Therapy Assistant Program, Bristol Community College, New Bedford, MA 02470, USA; E-Mail: christinehelfrich@gmail.com; 3 Department of Occupational Therapy, Boston University College of Health and Rehabilitation Sciences: Sargent College, Boston, MA 02215, USA; E-Mail: wjcoster@bu.edu; 4 Research Division at the Center for Psychiatric Rehabilitation, Boston University, Boston, MA 02215, USA; E-Mail: erogers@bu.edu

**Keywords:** homeless, environment, ICF, participation, housing

## Abstract

Community participation is an important goal for people who have experienced homelessness. The aim of this study was to use the International Classification of Functioning, Disability and Health (ICF) as a framework to examine factors associated with community participation among people who are homeless or recently housed through housing programs. Participants (n = 120) recruited from six housing placement and search programs completed measures of community participation (including productivity, social and leisure, and community-services-use domains), psychiatric and physical symptoms, functional limitations, and a demographic form. Multiple regression analyses were used to identify predictors of overall community participation and subdomain scores. Results suggested that cognitive and mobility limitations, relationship status, and housing status significantly predicted both overall participation and participation in productivity and social and leisure subdomains. Participants who were housed through housing programs, who had cognitive and mobility limitations, and who were single showed less community participation. The findings suggest that activity limitations and environmental and personal factors may need to be addressed in efforts to enhance community participation in this population.

## 1. Introduction

Homelessness is a prevalent societal problem in the United States. The National Alliance to End Homelessness [1] estimated that over 600,000 people experience homelessness every night with 54% being chronically homeless (*i.e.*, have been homeless continuously for at least one year or on at least four separate occasions in the last three years) [1].The majority of people who have experienced homelessness report severe hardship, disability and disease, which result in a substantial need for healthcare and social services [2]. 

Participation in the community is an ongoing challenge for this population. Individuals who are chronically homeless often experience disaffiliation from society, which is reflected in limited social relationships, unemployment, and limited access to welfare, religious, and social groups [3]. Even after becoming housed through support programs, their community participation may still show limited improvement [4]. As a result, these individuals may still remain marginalized and isolated, which affects their social functioning, health, and quality of life (QOL) [5].

The World Health Organization (WHO) International Classification of Functioning, Disability and Health (ICF) has defined participation as “involvement in life situations”, which is the result of a complex interaction among factors that include an individual’s body structure and function, activity, and personal and environmental factors [6]. Participation can be divided into two broad areas: home participation and community participation. Compared to participation in household activities, community participation usually requires different or even advanced capabilities, such as outdoor mobility and socializing with more and less familiar people [7]. Thus community participation can be defined more precisely as “active involvement in activities that are intrinsically social, and either occur outside of the home or are part of a non-domestic role, such as work, social (outside of the household), and other community roles” [7]. 

Research investigating community participation has indicated that a person’s involvement in the community may be lower when one or more of the following factors are present: more physical and psychiatric symptoms [8,9,10]; more posttraumatic symptoms [11]; limitations in mobility and daily activities [12]; lower cognitive ability [5]; lower interpersonal skills [13]; unmatched ethnicity with their community [14,15]; lower social economic status (SES) [16,17]; older age [9]; and less social support [18]. However, only a few studies investigated participation in people who are homeless or formerly homeless: Gulcur and colleagues [9] evaluated the influence of individual and program factors on community integration among people with psychiatric disabilities and with a history of homelessness; Yanos *et al*. [15] examined personal factors associated with community integration of mental health consumers living in supported housing. Even though these studies have looked at several elements of community participation among people who have experienced homelessness, none provide information on some potentially important factors such as activity limitations (e.g., cognitive and mobility limitations) and environmental variables (e.g., housing situation) associated with community participation. According to the ICF model, impairments, activity limitations, and personal and environmental factors should all be considered to have potential direct association with community participation [6]. Gaining a clearer understanding on how these factors are related to participation in the homeless population would help service providers develop better targeted efforts to assist homeless people to participate in the community, and increase policy makers’ awareness of the barriers and facilitators that may influence their community integration.

To address the gap in literature, we undertook this study to further our understanding of whether and how impairments, activity limitations, personal factors, and environmental factors were related to community participation among people who have experienced homelessness. The ICF was used as the framework for this study.

## 2. Methods 

This study was a cross-sectional study and was part of a longitudinal intervention study investigating the effect of a life skills intervention for chronically homeless individuals. The larger study included baseline, post-intervention, and two to three years of follow-up data collection. For the purpose of this study, we only used data collected at baseline (T1). Both the larger study and the current study were approved by a University’s Institutional Review Board. 

### 2.1. Participants

Participants were recruited from three housing search programs and three housing placement programs (including a Housing First Program and two Housing Stabilization Programs) in the greater Boston area. The Housing Search Programs were designed to help homeless individuals from the streets, shelters, or other homeless circumstances to obtain apartments and subsidized housing. After obtaining a home, the Housing Stabilization Programs helped individuals maintain housing through available support services such as life skills training. The Housing First Program provided congregate residences with supportive services. Participants in the Housing First and Housing Stabilization programs were housed at the time when they were recruited into this study, while participants in the Housing Search programs were still in the process of searching for a home.

A trained data collector coordinated with the providers of these programs to recruit participants and provide them the information of this study. The eligible participants had to meet the following inclusion criteria: (1) being at least 18 years old; (2) understanding English; (3) able to give informed consent; and (4) able to engage in 60-min group sessions and individual sessions. All of the participants were homeless and/or at risk for repeated homelessness. 

### 2.2. Measures

#### 2.2.1. Dependent Variable: Community Participation

The Community Participation Scale (CPS) was an objective measure of participation modified from the Client’s Assessment of Strengths, Interests, and Goals (CASIG) [19]. CASIG is a widely-used tool that evaluates social and instrumental functioning and treatment goals in the field of psychiatric rehabilitation. It includes nine functional subscales, of which four subscales are relevant to community participation: vocational, friends, leisure, and transportation. Items in these subscales ask about the occurrence of activities within the past three months with “yes/no” categorical responses. Wallace and Liberman [19] have provided evidence of the interrater reliability of all these items. These items have also shown moderate to strong test-retest reliability (r = 0.45–0.91) [19]. To cover other activities in which homeless people may participate, an additional subscale, “community,” was constructed by the research group for the larger study, such as going to the library, volunteering, attending a self-help group, and attending any spiritual or religious services, which were in the same response format as CASIG-functional items. An expert panel consisted of four measurement and rehabilitation experts reviewed the CASIG functional subscales along with the additional community subscale, and selected 26 items that fit the definition of community participation given in previous research [7]. These items covered three domains of community participation: productivity (e.g., have a paid job in the community), social and leisure (e.g., spend time talking to your friends), and community services use (e.g., receive primary health care).

Preliminary psychometric analyses were conducted to examine the scale properties of the 26-item CPS. The overall scale and two of the three domains: Productivity and Social-Leisure showed good internal consistency (Cronbach’s α > 0.80). However, the internal consistency of the community-services-use domain was weaker (Cronbach’s α = 0.64). The expert panel suggested eliminating these community-services-use items not only due to their weak item-correlations (r = 0.15–0.38) but also because of the concern regarding content validity (e.g., the health services use item “receive primary health care” raised a debate of whether these behaviors reflect homeless individuals’ “involvement” in the community or are behaviors that prevent them from participating in more diverse community activities). After eliminating the community-services-use items, the final scale covers the remaining two domains with 21 items. The final set of items demonstrated strong internal consistency (Cronbach’s α for the overall scale: 0.84; for the two domains: α = 0.80–0.81) and content validity (100% agreement between the experts regarding the content of the measure). Confirmatory factor analysis (CFA) was used to test the CPS with the two domains and showed good model-fit (comparative fit index (CFI) = 0.98, Tucker–Lewis Index (TLI) = 0.97, and root mean square error of approximation (RMSEA) = 0.046). The CPS was also moderately correlated with the quality of life (QOL) subscale of the CASIG (r = 0.47), which is consistent with literature suggesting that participation and QOL constructs are related [20]. More psychometric analysis results are published elsewhere [21].

#### 2.2.2. Predictor Variables 

**Figure 1:**
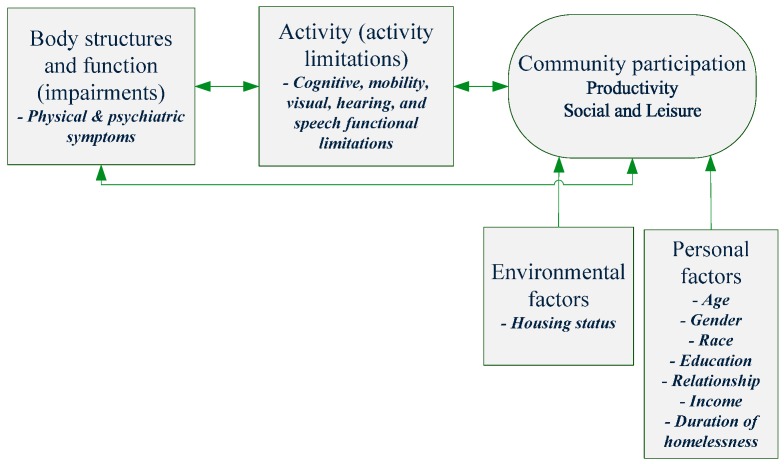
Model of potential factors related to community participation for people who have experienced homelessness.

The potential predictor factors, selected based on the ICF and literature review, are described in Figure 1. They included the following: psychiatric and physical symptoms, cognitive, physical, visual, hearing and speech functioning, demographics, homeless history, diagnosis, and housing status.

#### 2.2.3. Psychiatric Symptoms

We evaluated general psychiatric symptoms and traumatic symptoms with the Impact of Event Scale Revised (IES-R) and the Client’s Assessment of Strengths, Interests, and Goals (CASIG)-Symptom scale.

***The Impact of Event Scale Revised (IES-R).*** The Impact of Event Scale Revised (IES-R) is a Post-Traumatic Stress Disorder (PTSD) screen consisting of a 22-item self-report questionnaire with three subscales that measure the three diagnostic indicators of PTSD: intrusion (8 items), avoidance (8 items), and hyperarousal (6 items) [22]. Participants rate their level of distress using a 0 to 4 Likert scale. The total score of the IES-R is indicative of overall traumatic stress symptoms; higher scores indicate higher levels of symptoms. The IES-R has high internal consistency (Cronbach’s α = 0.96) and a high correlation with the PTSD Checklist, a standard measure to assess the DSM-IV symptoms of PTSD (*r* = 0.84). The subscales also demonstrate strong internal consistency (Cronbach’s α = 0.87–0.94) [23]. 

***CASIG-Symptom Scale.*** The CASIG-Symptom scale evaluates six symptoms (delusions/thought disorder, hallucinations, anxiety, depression, suicidal intentions, and mania) with seven self-reported dichotomous items. The reliability and validity of this symptom scale has been evaluated in community-dwelling and inpatient individuals with severe mental illness. The internal consistency and test-retest reliability are good (α = 0.71–0.76, *r* = 0.71–0.82). The inter-rater reliability (agreement between interviewee and observer) of this scale is low (*r* = 0.26–0.32), with interviewees reported more symptoms than the observers.

#### 2.2.4. Physical Symptoms

Physical symptoms were measured by an open-ended question from the Survey of Income and Program Participation (SIPP) topical questionnaire (2008), “Do you have any medical conditions? If so, please list all physical and psychiatric conditions” [24]. The total number of physical health conditions was counted by the researchers as a continuous indicator, ranging from 0 to 7.

#### 2.2.5. Cognitive Ability 

Two assessments were used to measure cognitive ability:

***The Allen Cognitive Levels Screen—2000 (ACLS).*** The Allen Cognitive Levels Screen—2000 (ACLS) is a performance-based evaluation of functional cognition, including the ability to process and utilize information and the capacity to learn or relearn skills as demonstrated with a leather lacing task. The Screen also predicts the level of assistance that an individual will need to perform routine tasks and how he/she will perform in novel situations. Results yield an ordinal score on a 25-point scale, ranging from 0 to 5.8. A higher score represents a higher cognitive level. The inter-rater reliability of the ACLS is high (*r* = 0.99) [25]. 

***CASIG-Cognitive Difficulty Scale*.** The CASIG-Cognitive difficulty scale includes six dichotomously-scored items about self-perceived difficulties in memory, attention and problem solving. In people with severe mental illness the scale showed a high internal consistency (Cronbach’s α = 0.76), fair test-retest reliability (*r* = 0.40), and low client-clinician rating agreement.

#### 2.2.6. Other Functional Limitations

To measure other functional limitations, five single item questions from the SIPP topical questionnaire Functional Limitation section were used to identify physical, vision, hearing, and speech limitations (e.g., “do you have difficulty hearing what is said in a normal conversation with another person?”).

#### 2.2.7. Personal and Environmental Factors 

Information on personal and environmental factors was collected using the Survey of Income and Program Participation (SIPP) topical questionnaire and a supplemental personal history and demographic form designed for the larger study [24]. The information includes demographics (*i.e.*, gender, age, race, marital status, income, education, relationship status and children), homelessness history, housing status, self-reported diagnosis of mental illness, substance use and disability, and health insurance.

### 2.3. Statistical Analyses

All statistical analyses were performed using SAS software version 9.3. No significant departures from normality were found in the variables. To identify significant factors related to community participation, we first examined bivariate associations between community participation and independent variables using Pearson’s correlation coefficient and one-way ANOVA. Variables associated (at least *p* < 0.10) with community participation were then included in multiple linear regression models predicting overall community participation and the two subdomain scores (productivity and social/leisure). Considering the number of the potential variables, the stepwise selection was applied in the regression analyses to identify a model with the best fit. Some participants were dropped from the stepwise selection process due to missing data on one or more of the variables that are not included in the final model. Therefore we re-ran the multiple regression model without stepwise selection and with the variables identified in the final model. This model re-fitting approach can be employed to reduce concerns about the loss of the sample size and power due to subjects who were dropped out of the analysis.

## 3. Results 

One hundred and forty-nine people were recruited into the study. Thirteen subjects were unreachable after completing the consent form; one subject did not complete baseline assessments; 15 subjects withdrew from the study after consenting to participate due to health issues, housing crises, employment, being imprisoned, or other personal issues. Thus, a final sample of 120 remained for analysis.

Table 1 displays the demographic characteristics of the participants. Of the 120 participants, 40 affiliated with the Housing Search program were currently homeless, while 80 in the Housing Stabilization program or Housing First program were currently housed in affordable housing and pursing stable tenancies. Participants’ duration of homelessness averaged 5.31 years (range 7 days to 31 years). Most of the participants were living in poverty (71% had an annual income of less than $10,000), and their most frequently reported income sources were government subsidy and social welfare. The majority of the participants was single and had no children living with them.

**Table 1:** Characteristics of the sample (n = 120).

Characteristics	N (%)	Mean (SD) (years)
Age		48.76 (10.22)
Gender		
Male	74 (61.67%)	
Female	46 (38.33%)	
Race		
White	55 (45.83%)	
African-American	52 (43.33%)	
Hispanic/Asian/Other	13 (10.83%)	
Education		
Less than high school	19 (16.10%)	
High school graduate or GED	41 (34.75%)	
Some college	34 (28.81%)	
College graduate or more	24 (20.34%)	
Duration of homelessness (days)		5.31 (6.31)
>1 year	94 (81.03%)	
<1 year	22 (18.97%)	
Housing programs		
Housing search	40 (33.33%)	
Housing placement		
(Housing first)	34 (28.33%)	
(Housing stabilization)	46 (38.33%)	
Relationship status		
Married, life partner, in a relationship	24 (20.17%)	
Single, widowed, separated, divorced	95 (79.83%)	
Living with children	2 (1.67%)	
Disability	83 (69.75%)	
Diagnosis of mental illness	77 (64.71%)	
Substance abuse	69 (60.00%)	

Note: Totals < 120 indicate missing data.

The bivariate analyses shown in Table 2 indicated that none of the impairment variables were correlated with community participation at a significant level (*p* < 0.05). On the other hand, community participation was significantly correlated with multiple activity limitation variables, including cognitive (ACL and CASIG-cognitive), mobility, visual, and hearing limitations. Those without activity limitations showed higher participation than those with limitations. Several personal and environmental factors including gender, age, homelessness duration, relationship status, and housing status were also significantly correlated with community participation. Those who were not housed or experienced shorter homelessness duration reported higher scores on overall community participation. Scores of Productivity were higher in people with higher cognitive function, males, younger people, those with no home, and those who experienced shorter homelessness duration. Social and leisure participation was significantly correlated with cognitive, mobility, and speech limitations and housing status.

**Table 2:** Bivariate analysis (one-way ANOVA/ Pearson’s correlational analysis) results of community participation (CP) and the independent variables.

		Overall CP	Productivity	Social and Leisure
**Impairment Variables**				
IESR		r = −0.07	r = 0.16 ^†^	r = −0.16 ^†^
Psychiatric symptoms (CASIG)		r = 0.17	r = −0.06	r = −0.13
Number of physical symptoms		r = −0.06	r = −0.05	r = −0.13
**Activity Limitation Variables**				
ACL		r = 0.20 *	r = 0.29 **	r = 0.11
Cognitive difficulty (CASIG)		r = −0.19 *	r = −0.01	r = −0.23 *
Mobility limitation	Yes	7.13 **	0.97	6.16 **
	No	9.49	1.51	7.98
Visual limitation	Yes	7.05 *	0.44	6.21 ^†^
	No	8.92	1.84^†^	7.48
Hearing limitation	Yes	6.42	0.67	5.75 ^†^
	No	8.71 *	1.36 ^†^	7.34
Speech limitation	Yes	6.64 ^†^	1.14	5.50 *
	No	8.61	1.24	7.37
**Personal and Environmental Variables**				
Gender	Male	8.49	1.51 *	6.97
	Female	7.87	0.76	7.11
Age		r = −0.17 ^†^	r = −0.20 *	r = −0.12
Race	White	8.75	1.13	7.62
	Black	8.12	1.33	6.79
	Other	6.33	1.67	4.67
Education	Less than high school	9.00	1.58	7.42
	High school graduate or GED	7.00	0.85	6.15
	Some college	9.68	1.79	7.88
	College graduate or more	7.50	0.75	6.75
Homeless duration	>1 year	7.84 *	1.04 *	6.80 ^†^
	<1 year	10.55	2.18	8.36
Relationship status	Married/life partner/in a relationship	9.75 ^†^	1.75 ^†^	8.00
	Single/widowed/separated/divorced	7.79	1.03	6.76
Mental illness	Yes	7.86	1.12	6.74
	No	8.93	1.40	7.52
Substance use	Yes	8.83	1.43	7.39
	No	7.70	1.00	6.70
Housing status	Housed through housing programs	7.24	0.83 *	6.41 *
	Homeless	10.28 **	2.03	8.25

Notes: Values shown are mean scores of the CPS or correlation coefficient (r); ^†^ *p* < 0.1, * *p* < 0.05, ** *p* < 0.01.

Based on the final model of stepwise selection, we refit the retained variables into a multiple linear regression model. The results of the stepwise regression and refitted model are shown in Table 3. Overall community participation was significantly predicted by housing status and mobility limitation (*p* < 0.05). Productivity was significantly predicted by cognitive limitation, relationship status, and housing status. Social and leisure participation was predicted by cognitive limitation, mobility limitation, and housing status. Of all these variables, housing status appears to be the strongest and most consistent predictor. Those who were currently housed showed lower participation in all areas than those who were currently homeless.

**Table 3:** Multiple regression model of community participation and its subdomains (N = 120).

N	Overall CP	Productivity	Social and Leisure
	118	105	118
R^2^	0.16	0.22	0.17
F-value (** *p <* 0.01)	7.00 **	9.23 **	5.60 **
Variables	standardized β (*p-*value)		
Cognitive difficulty	−0.16 (*0.06*) ^†^	--	−0.19 (*0.03*) *
ACL	--	0.26 (*0.006*) **	--
Mobility limitation	−0.19 (0.03) *	--	−0.20 (0.03) *
Speech limitation	--	--	−0.16 (0.07) ^†^
Relationship status	--	0.19 (*0.03*) *	--
Housing status	−0.25 (*0.004*) **	−0.27 (*0.003*) **	−0.18 (*0.04*) *

Notes: Values shown are standardized beta coefficients (*p*-value); -- means the variable was not included in the model; ^†^ *p* < 0.1, * *p* < 0.05, ** *p* < 0.01.

## 4. Discussion

Drawing on the ICF conceptual model, this study identified significant associations between community participation and multiple activity limitations, personal factors, and environmental factors. Of these factors, housing status demonstrated the strongest impact on community participation. Previous research suggested that stable housing offers a secure base for individuals to develop meaningful activities, social network, and friendships [26]. Numerous qualitative and quantitative studies have indicated that people who had experienced homelessness but were placed in permanent homes demonstrated better global functioning, health, quality of life, and community integration compared to those who were in unstable accommodations [27]. However, our results contradict these findings and conclusion and indicate that participants who were currently homeless demonstrated more community participation than those who were housed through housing programs. In our sample, those who were housed were less likely to engage in work, social, and leisure activities. One possible explanation for this contradiction may be that unhoused participants were not able to spend much time in their temporary accommodations, and therefore must seek opportunities in the community, thereby increasing the appearance of community participation. Accommodations such as transitional housing or emergency shelters are temporary and structured to ensure that people do not stay for the long-term [28]. Some shelters may only provide overnight accommodation, and residents have to leave the shelter during the day. Even if individuals can stay in temporary housing during the day, many homeless people may not be motivated to do so due to their negative perception of the shelters. Several qualitative studies have indicated that homeless individuals find shelters to be unclean, unsecure, uncomfortable, and lacking privacy [27,29,30]. In addition, many individuals express dissatisfaction with staff and regulation policies of their transitional housing [30,31]. 

People who live in permanent housing would be expected to have more space and time to do things at home, such as preparing meals, grooming, doing chores, or simply relaxing. Engaging in such activities within the home would then not necessitate as much community participation activity. Those who are not housed, however, may need to spend more time in the community searching for food, shelter, and other resources. For example, they may go to local churches or functions for free meals, or go to a local library to get warmth and rest. They may also spend more time networking with other homeless people to share food, exchange information about survival, and talk about personal problems [32]. These activities mimic community participation but many appear to be driven by a lack of housing during the day and not truly participatory. Further investigation will be needed to examine differences in the adequacy of community activity engagement between homeless and housed individuals, and to explore the actual reasons they engage in community activities.

Activity limitations, particularly cognitive and mobility limitations, also showed significant impact across all regression models. Individuals with fewer functional activity limitations reported more participation in work, social, and leisure activities. On the other hand, none of the impairment variables were significantly associated with participation in the multivariate regression models. This finding is consistent with the conceptualization of participation in the ICF, which proposes that the impact of impairment is indirect, by way of its influence on activity performance. Previous research has supported this hypothesis. For example, Jette *et al*. [12] found that regardless of the type and severity of impairment, physical and cognitive activity limitations were the best predictors of variance in community participation in post-acute rehabilitation patients. Studies of community-dwelling older adults also report that activity limitations have a direct association with participation and largely explain the correlation between impairment and participation [16]. Results of the current study replicate this finding in people who have experienced homelessness. 

Relationship status was a significant factor particularly related to Productivity. Our results suggested that those with a spouse/life partner/relationship reported participating in more productive activities. This finding is consistent with those from other studies investigating social support and participation in people with disabilities or living in poverty. A large body of research indicates that positive social support from significant others predicts higher likelihood of employment [33,34,35]. People who have experienced homelessness tend to have fewer and more tenuous social networks and often experience isolation and loneliness [36]. Thus an intimate relationship is very likely to be one of the main sources of social support, which has a protective effect on multiple health outcomes in homeless people as well as the general population [37,38].

Our study has several limitations. First, aside from limitations in the small sample size, the generalizability of this study may be limited to the Boston area, where the distribution of community resources such as transportation and entertainment settings may be completely different from those in a rural area. Investigations from other urban centers as well as more suburban and rural settings are needed to address this question.

Second, the study was a part of a larger study and we were restricted to the measures that were available to assess variables of interest. For example, some personal and environmental factors, such as social skills, length of time in current housing, culture, and stigma that may also influence community participation were not included in the analyses [13,15,39]. Aside from these variables that were not collected for the larger study, the variables we examined in this study have covered most of the ICF components. In future studies of the homeless population, the relation between community participation and more environmental variables should be examined with a more subtle design and a larger sample.

Furthermore, the CPS that we used in this study only asked about the occurrence of activities, which just summarizes the variety of activities in which the individuals participate. In recent literature, participation has been recognized as a multidimensional concept, which needs to be measured with different approaches to reflect the construct [40]. The lack of a good measure of community participation that addresses multiple dimensions has been a challenge not only to homelessness research but also to disability and rehabilitation research [7,40]. To expand coverage of the construct of participation, an instrument that examines more dimensions of participation, such as frequency, satisfaction, and desire for change, is recommended. Moreover, although the community-services-use subscale was eliminated from the CPS based on results of the psychometric testing and the experts’ suggestions, more discussion may be needed regarding whether community activities such as shopping at a store, running errands, and going to see a healthcare professional should be included in a measure of community participation. Salzer *et al*. [41] found that going shopping, using public transportation and going to run errands were areas that most people with serious mental illnesses (SMI) identified as important to them. However, in a later measurement validation study, Chang *et al*. [42] found that these items showed extremely skewed frequency distribution, as a result of which they decided not to include them in a community participation measure. Determining how best to reconcile “clinical importance” and “statistical concerns” to create a sound measure of community participation is an on-going challenge that needs further discussion. 

Finally, the cross-sectional nature of this study limits our ability to draw conclusions regarding causal relationships between predictive factors and community participation. Future longitudinal research is recommended to further investigate possible causal relationships as well as change over time in community participation. 

## 5. Conclusions

To our knowledge, this study is the first study using the ICF model to identify factors and shed light on potential determinants associated with community participation among people who have experienced homelessness. Several implications can be drawn from the findings. First, aligned with the ICF model, activity limitations had a direct association with community participation. This finding suggests that if the rehabilitation goal is to improve overall community participation, interventions may need to focus more on enhancing activity capability such as cognitive and mobility functioning rather than on lessening illness-related symptoms. Second, more attention may need to be paid to people who are housed since they showed less involvement in the community. Although having a stable living place is, overall, a positive step, service providers should not regard it as the exclusive indicator of recovery. Instead, they need to evaluate whether those who have transitioned from homelessness need assistance and services to progress toward sustainable levels of independence and community participation. Further investigation of the actual reasons behind the complex participation behaviors across subgroups will help the field to better understand and address the needs of this population. 
